# The patent foramen ovale may alter migraine brain activity: A pilot study of electroencephalography spectrum and functional connectivity analysis

**DOI:** 10.3389/fnmol.2023.1133303

**Published:** 2023-03-07

**Authors:** Xiangyu Lei, Meng Wei, Yi Qi, Liang Wang, Chenyu Liu, Yichen Guo, Yue Xu, Xiangqi Cao, Rui Liu, Guogang Luo

**Affiliations:** Department of Neurology, The First Affiliated Hospital of Xi’an Jiaotong University, Xi’an, Shaanxi, China

**Keywords:** migraine, patent foramen ovale, EEG, spectrum power, phase lag index

## Abstract

**Background:**

A link has been shown between patent foramen ovale (PFO) and migraine, particularly migraine with aura. However, it is unknown if PFO might cause migraine by altering cortical excitability and neural network, which may lower the threshold of cortical spreading depression (CSD). This study aims to compare the spectrum power and functional connectivity of the alpha and beta bands of electroencephalography (EEG) across migraine patients with and without PFO.

**Methods:**

Thirty-five migraine patients with PFO (PFO +), 35 migraine patients without PFO (PFO –) and 20 PFO patients without migraine (control) were enrolled in this cross-sectional analysis. 19-channel EEG was recorded for all patients under resting state and intermittent photic stimulation. Power spectrum density (PSD) and phase lag index (PLI) of alpha and beta bands were then calculated and compared between the three groups.

**Results:**

During photic stimulation, the beta band PSD at the occipital area was substantially higher in PFO + migraine patients compared to PFO-migraine patients (*p* < 0.05, Bonferroni corrected). Subgroup analysis showed that both migraine with and without aura patients with PFO had increased PSD in the alpha and beta bands at the occipital region during photic stimulation (*p* < 0.05, Bonferroni corrected). Meanwhile, the beta band PLI during photic stimulation was significantly elevated (adjusted *p* = 0.008, utilizing the network-based statistic technique) in PFO + group compared to PFO-group. Furthermore, although failed to pass the correction, the beta band power in the occipital area during photic stimulation at 20 Hz on O1 (*R* = 0.392, *p* = 0.024) and O2 channel (*R* = 0.348, *p* = 0.047) was prone to positively correlated with MIDAS score, and during photic stimulation at 12 Hz on O2 channel (*R* = 0.396, *p* = 0.022) and 20 Hz (*R* = 0.365, *p* = 0.037) on O1 channel was prone to positively correlated to HIT-6 score in PFO+ migraineurs, whereas no similar correlation was found in the PFO-group patients.

**Conclusion:**

The outcomes of this investigation suggested that PFO may change the cortical excitability in the occipital lobe of both migraineurs with and without aura. Meanwhile, the beta band PSD on the occipital area during photic stimulation might be an objective measure of severity in migraineurs with PFO.

## 1. Introduction

Migraine impacts almost 1 billion people’s healthy lives worldwide, and it is the sixth most prevalent condition and the second leading cause of disability (Lipton et al., 2007; (WHO) TWHO, 2016, 2017). Numerous studies have demonstrated that migraine, particularly migraine with aura (MA), is strongly comorbid with patent foramen ovale (PFO), a fetal circulatory remnant that affects around 20–30% of the general adult population (Liu et al., 2020; Ahmed and Sommer, 2021; Mojadidi et al., 2021). The most commonly recognized theory is that PFO allows some kind of vasoactive substances or paradoxical embolus to go directly to arterial circulation and reach the brain, causing the migraine phenomenon (Kumar et al., 2019). However, the pathophysiological relationship and correlation between migraine and PFO is far from clear.

Previous research has suggested that cortical spreading depression (CSD) is the fundamental physiological etiology of migraine aura (Pietrobon and Moskowitz, 2013). Nozari et colleagues investigated the relationship between PFO and migraine with aura in mice by introducing particulate or air microemboli into the carotid circulation and discovered that transitory microvascular blockage might trigger CSD without infarction (Nozari et al., 2010). These findings suggested that PFO might cause CSD by paradoxical embolism, resulting in a migraine aura event. In a clinical study concentrating on MA patients with large PFO, Sevgi et al. discovered that the electroencephalography (EEG) power was considerably raised in MA patients with large PFO but not in MA patients without PFO or PFO patients without migraine after microbubbles infusion (Sevgi et al., 2012). In addition, a recent study of MA patients with PFO confirmed that these patients had elevated platelet activation indicators that decreased to levels comparable to healthy controls after PFO occlusion (Trabattoni et al., 2022). These investigations lead us to believe that platelet aggregation and paradoxical embolism might induce migraine attacks and that migraineurs with PFO are more vulnerable to transitory hypoxia, implying that PFO may impact migraine cortical excitability, which is currently unknown.

To gain a better understanding of the pathophysiological relationships between PFO and migraine, researchers must look into the persistent effect of PFO on migraine brain activity. As an objective record of brain activity with a high temporal resolution, EEG has played an important role in detecting subtle brain dysfunctions. EEG spectrum analysis revealed that migraineurs’ alpha rhythm power is increased in the occipital region (Clemens et al., 2008). After visual contrast detection, the amplitude of alpha-band oscillations was higher in the occipital region in migraine patients compared with controls (O'Hare et al., 2018). EEG functional connectivity studies have also revealed that during photic stimulation, migraineurs have increased alpha band synchronization between the basal region and the scalp (Angelini et al., 2004). However, previous studies did not consider the effect of PFO on migraine.

The phase lag index (PLI) can provide a reliable estimate of phase synchronization that is resistant to the presence of common sources. The PLI performed better in detecting true connectivity and was more resistant to the common sources problem (Stam et al., 2007). This study aims to: (1) determine whether PFO has long-term effects on migraine brain activity, especially the activity of the occipital region, by detecting differences in spectrum power and PLI-based functional connectivity between migraine patients with and without PFO; and (2) determine the relationship between abnormal brain activity and migraine clinical characteristics in PFO + patients.

## 2. Materials and methods

### 2.1. Subjects

Migraine patients who meet the International Classification of headache disorders III [ICHD-III; Headache Classification Committee of the International Headache Society (IHS), 2018] criteria for episodic migraine were enrolled in this study from the Headache Clinic of the Department of Neurology, The First Affiliated Hospital of Xi’an Jiaotong University from September 2020 to October 2021. This study included patients aging from 18 to 65, with at least 1 migraine headache attacks per month in the last 3 months and at least 1 year of course, no previous regular migraine prophylaxis and written consent. Meanwhile, we also recruit patients with PFO but without migraine as control. Patients with other neurological disorders which could influence brain activity (e.g., stroke, epilepsy, and psychological disease) would be excluded. The present study was approved by the Ethics Committee of Xi’an Jiaotong University First Affiliated Hospital (XJTU1AF2020LSK-226).

Clinical information including basic information and headache characteristics was collected. Headache characteristics include migraine course, attack frequency (number of attacks per month experienced in the last 3 months), headache days per month (in the last 3 months), duration of headache attacks, headache severity (*via* Visual analogue score, VAS, from 0 to 10), Migraine Disability Assessment Score (MIDAS; Stewart et al., 1999), and Headache Impact Test (HIT-6; Kosinski et al., 2003). PFO was screened by TCD and identified by cTTE: micro-bubbles appear in the left ventricle within five cardiac cycles after the Valsalva maneuver. Patients with inconsistent TCD and cTTE results were rechecked for cTTE when consent was obtained. Patient grouping is determined by final review of cTTE results. The PFO was classified into four grades according to the cTTE: (Lipton et al., 2007) grade 0, no shunt (no bubble); [(WHO) TWHO, 2016] grade 1, small shunt [1–10 bubbles; (WHO) TWHO, 2017] grade 2, middle shunt (11–30 bubbles); (Ahmed and Sommer, 2021) grade 3, large shunt (> 30 bubbles or full of bubbles in the left atrium). All patients were then classified into two groups: migraine with PFO group (PFO +) and simple migraine group (PFO –). Following that, EEG was acquired for all patients.

### 2.2. Electroencephalography data acquisition

Electroencephalography data were recorded for all patients during the interictal phase (at least 24 h before and after the pervious/next attack) by 19 scalp electrodes according to the international 10–20 system (Fp1, Fp2, F3, F4, C3, C4, P3, P4, O1, O2, F7, F8, T3, T4, T5, T6, Pz, Cz, Fz, with impedance below 5,000 Ω), with average reference, using Nihon konden-1200c EEG system. Four additional electrodes for ECG and EOG recording. Fpz served as the ground electrode. Signals were filtered to 0.5–70 Hz at a sampling rate of 500 Hz. All patients had their EEGs recorded while resting and during photic stimulation.

Resting state EEG was recorded when patient is awake, eyes closed and relaxed. Photic stimulation was presented successively at 1, 2, 4, 6, 8, 10, 12, 14, 16, 18, 20, 60, 50, 33, 30, and 25 Hz frequency with Nihon Konden-LS 703A photic stimulator. 10-s (s) stimulus for each stimulus frequency, followed with a 7-s rest period, 3 times for each patient. The distance from the stimulator to nasion was 20 cm. All EEG data was exported to a local database in the Department of Neurology, Xi’an Jiaotong University First Affiliated Hospital, for storage and future analysis.

### 2.3. EEG data preprocessing

Electroencephalography data preprocessing are performed through EEGLAB v14.1.2 (Delorme and Makeig, 2004; Swartz Center for Computational Neuroscience, University of California, San Diego, CA, United States), running under MATLAB R2020a (Mathworks, Sherborn, MA, United States). EEG raw data were re-referenced to average, with a band-filter to 0.5–70 Hz and notch filter at 50 Hz, and epoched between-1 and 10 s relative to the onset of each photic stimulation. EEG data at resting state is also cut into 10 s epochs for at least 30 s records free of artifacts. All epochs were visually inspected for noisy data which were then rejected. The mean activity in the 1 s period before stimulus onset was subtracted from the rest of the epoch for baseline correction. Independent component analysis (ICA; Bell and Sejnowski, 1995) was then used to identify and remove artifacts such as eye blinks, eye movements and heartbeats. The pre-processed EEG data were subsequently subjected to power spectrum and functional connectivity calculations. As mentioned before, previous studies have found that EEG changes during photic stimulation in migraine patients are mainly in the occipital region, so the subsequent calculation and comparison of power spectra and functional connectivity in this study also focus on changes in the occipital region.

### 2.4. Power spectrum analysis

Electroencephalography power spectrum density (PSD) was calculated using Brainstorm (Tadel et al., 2011) toolbox running under MATLAB, which is documented and freely available for download online under the GNU general public license,^1^ by means of Fast Fourier Transform (FFT), with a default of 1 s Hamming window length and 50% overlap. PSD of alpha (8–13 Hz) and beta (14–30 Hz) band was estimated for all 19 recording channels from 0 to 10s of all epochs. Since this study focuses on the brain activity at the occipital region, the PSD values of the O1 and O2 channels were compared between groups.

### 2.5. Functional connectivity analysis

CSDtoolbox running under MATLAB was used to compute scalp surface Laplacian for all EEG records firstly^2^ (Kayser and Tenke, 2006a,b). Then we evaluate the phase lag index (PLI) between all 19 electrodes as functional connectivity. PLI is the most commonly used phase interaction measure. The range of PLI is between 0 and 1. A PLI of zero indicates either no coupling or coupling with a phase difference centered around 0 mod π. A PLI of 1 indicates perfect phase locking at a value different from 0 mod *π* (Stam et al., 2007). Here, we focus on PLI on alpha and beta bands between the occipital region and other brain regions and compare them between the two groups. The PLI was visualized with the BrainNet Viewer^3^ (Xia et al., 2013).

### 2.6. Statistical analysis

Statistical analysis was performed using SPSS v25.0 (IBM Corp, Armonk, NY, United States) and dNBS toolbox of MATLAB. Comparison of demographics and headache characteristics between groups used one-way analysis of variance based on data normality and Chi-square tests for categorical variables with a statistical threshold *p* < 0.05. For comparison of PSD between groups, general linear model was used after adjusting for age and gender. The Bonferroni post-hoc correction for multiple comparisons was applied, yielding an adjusted *p* < 0.0083(0.05/6) for three groups and two channels.

We use the network-based statistics (NBS) method for comparison of PLI between groups *via* dNBS toolbox. NBS is a validated method for performing statistical analysis on large networks. Numerous studies have used this method to identify connections and networks between groups. One-way analysis of covariance was used to compare PLI of three groups after adjusting for age and gender using NBS method, *post-hoc* analysis was applied using Bonferroni correction with an adjusted *p* < 0.0167(0.05/3) for three groups.

Partial correlation analysis was used to investigate the relationships between PSD/PLI and headache characteristics in migraine with PFO patients adjusted for age and gender.

## 3. Results

### 3.1. Clinical characteristics

This study included 70 episodic migraine patients and 20 PFO patients without migraine. In migraine patients, 35 cases had PFO and the other 35 did not (Table 1). There was no significant difference in the distribution of the groups’ age, gender, educational qualifications, and other clinical characteristics (*p* > 0.05).

**Table 1:** Clinical characteristics of three groups.

	Migraine with PFO group	Migraine without PFO group	Control group	Sig.
N	35	35	20	–
Age (years)	35.03 ± 10.96	34.26 ± 10.73	38.40 ± 13.21	0.505
male/female	4/31	8/27	7/13	0.110
Educational qualifications (years)	13.83 ± 4.39	13.77 ± 3.70	14.45 ± 2.91	0.786
With aura/without aura	11/24	7/28	–	0.274
Course (years)	9.69 ± 8.20	12.99 ± 8.34	–	0.100
Attach frequency (attaches/month)	3.08 ± 2.87	4.05 ± 3.53	–	0.211
Attach days (days/month)	3.71 ± 2.99	4.93 ± 4.62	–	0.193
VAS^*^	6.26 ± 1.54	6.77 ± 1.70	–	0.189
Duration (hours)	18.00 ± 20.24	17.30 ± 16.53	–	0.875
HIT-6^*^	55.83 ± 8.86	58.11 ± 5.03	–	0.189
MIDAS^*^ (total days)	7.26 ± 5.93	10.74 ± 10.76	–	0.100

* VAS: visual analogue scale; HIT-6: headache impact test-6; MIDAS: migraine disability assessment scale.

Continuous variables are expressed as mean ± SD.

All 35 patients in PFO + group had a large right-to-left shunt. Besides, there were 30 patients in PFO+ group had a right-to-left shunt at resting state.

We further separated patients by migraine subtype for subgroup analysis to clarify the effect of PFO on the EEG-related indicators of different subtypes of migraine. The clinical characteristic of subgroups is shown in Supplementary Tables 1 and 2. There were no component differences in any of the indicators, except for the gender of the MO patients between PFO + and PFO– (*p* = 0.008).

### 3.2. Spectrum power differences between groups

**Figure 1:**
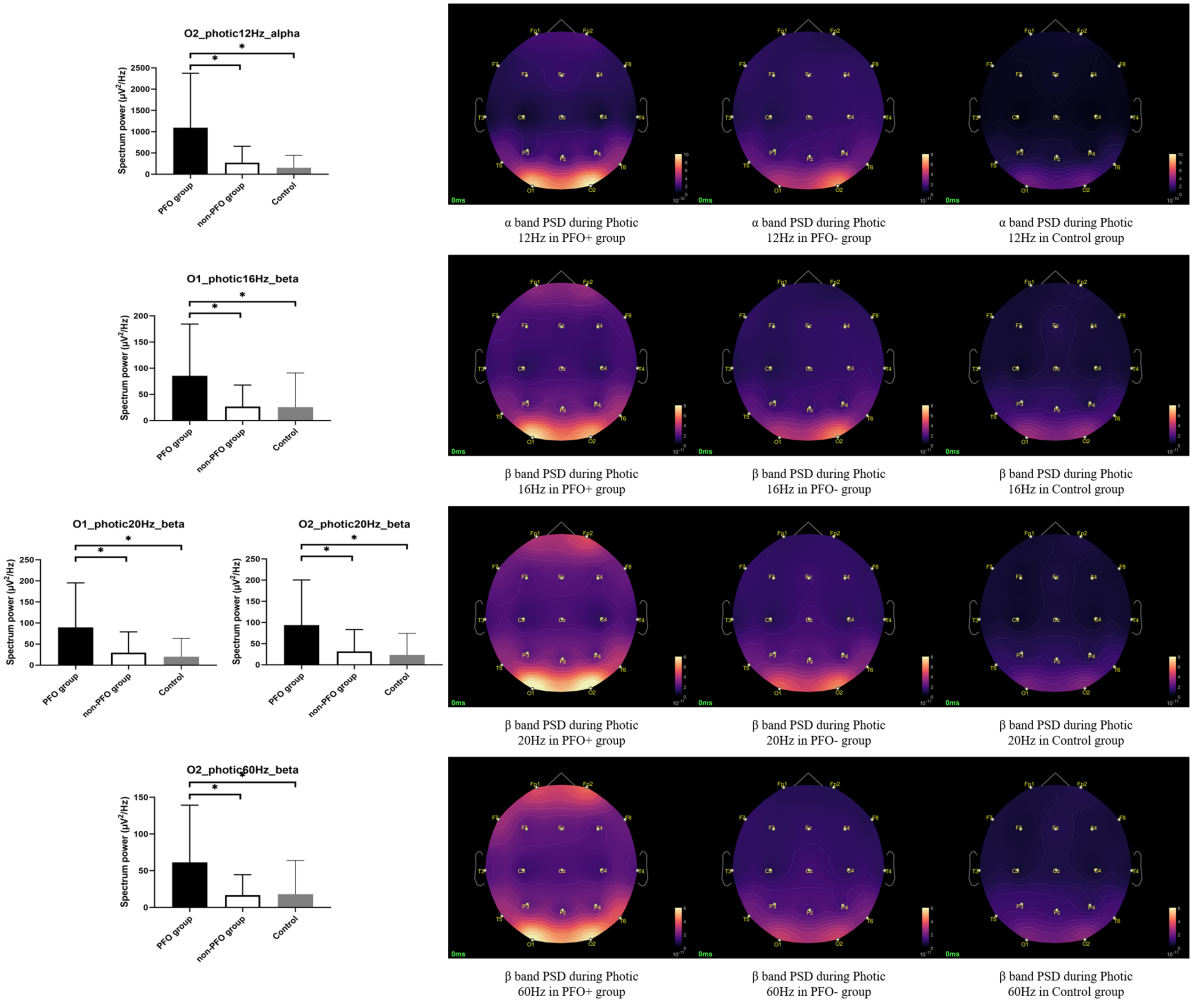
Comparison of alpha and beta band power spectrum density (PSD) on the occipital region during photic stimulation among patent foramen ovale (PFO) + group (*n* = 35), PFO-group (*n* = 35) and Control group (*n* = 20). The alpha band PSD on the occipital region was significantly increased in the PFO + group during photic stimulation at 12 Hz, and beta band PSD on the occipital region was significantly increased during photic stimulation at 16, 20, and 60 Hz, as shown by box graphs. The PSD topography on the right side provides a more intuitive representation of the distinction between the two groups. ^*^ adjusted *p* < 0.05.

PFO + group has significantly higher PSD of the beta band on the O1 channel during photic stimulation at 16 Hz compared to PFO-group and control group (*p* = 0.008 for comparison between PFO + and PFO –, *p* = 0.008 for comparison between PFO + and control). During photic stimulation at 20 Hz, the PSD of the beta band on both O1 and O2 channel is significantly increased in the PFO + group (*p* = 0.006 for comparison between PFO + and PFO –, *p* = 0.002 for comparison between PFO + and control). Furthermore, the PSD of the beta band on the O2 channel during photic stimulation at 60 Hz is significantly increased in the PFO + group (*p* = 0.008 for comparison between PFO + and PFO –, *p* = 0.007 for comparison between PFO + and control). As for the alpha band, only during photic stimulation at 12 Hz, the PFO + group showed an increased alpha band PSD compared to other two groups (*p* = 0.001 for comparison between PFO + and PFO –, *p* = 0.001 for comparison between PFO + and control, Figure 1). There are no significant differences in PSD on alpha and beta bands of O1 and O2 channels between PFO + group and PFO – group under resting-state.

**Figure 2:**
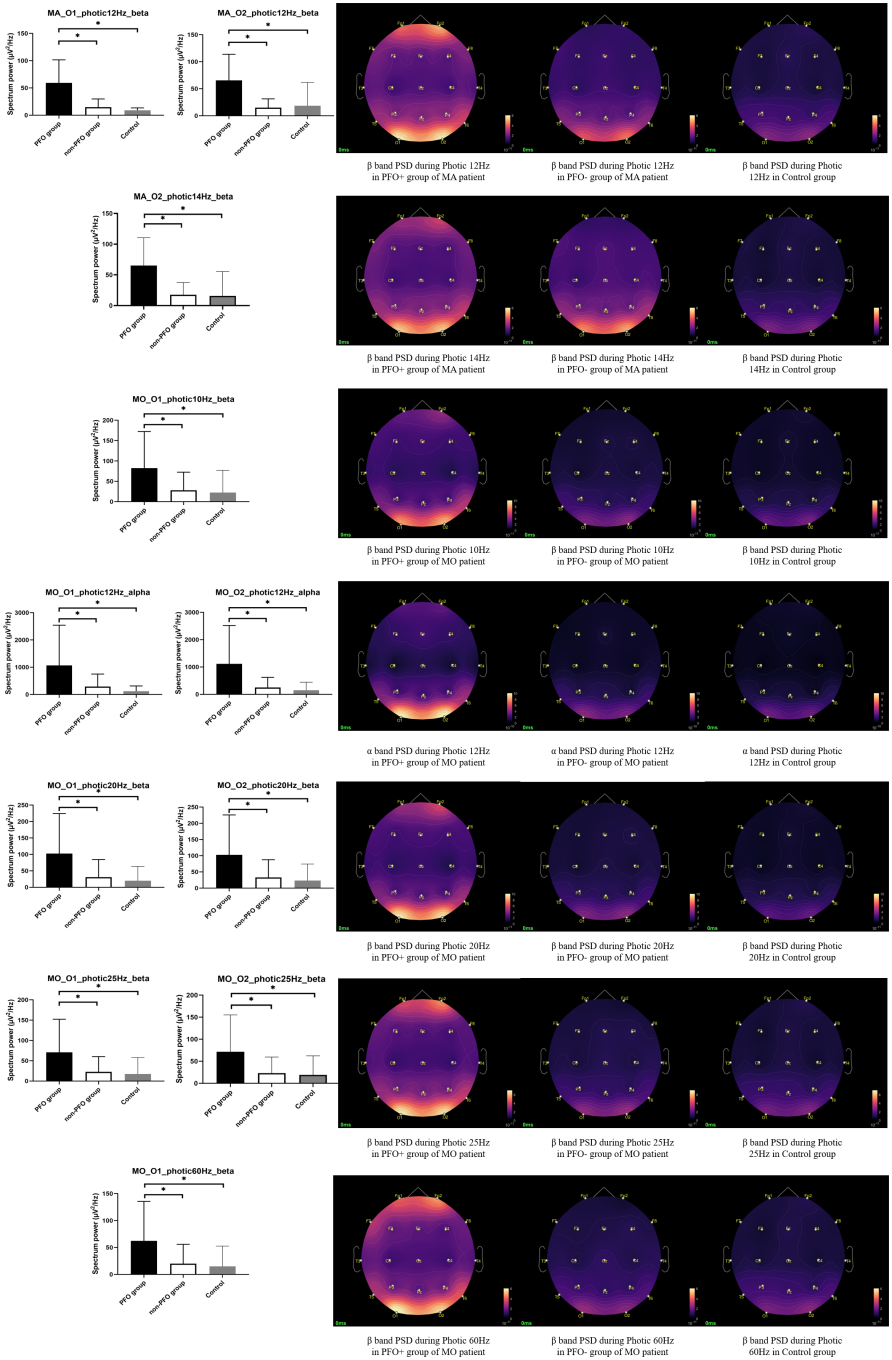
Subgroup analysis results of alpha and beta band PSD on the occipital region during photic stimulation. In migraine with aura (MA) patients, the PFO + group (*n* = 11) showed an increased beta band PSD during photic stimulation at 12 and 14 Hz compared to PFO-group (*n* = 7) and Control group. Whereas in MO patients, the PFO + group (*n* = 24) presented higher alpha band PSD during photic stimulation at 12 Hz and higher beta band PSD during photic stimulation at 10, 20, 25, and 60 Hz. The PSD topography on the right side provides a more intuitive representation of the distinction between the two groups. ^*^ adjusted *p* < 0.05.

The results of the subgroup analysis showed that MA patients with PFO + showed an increased beta band PSD during photic stimulation at 12 Hz on both O1 (*p* = 0.004 for comparison between PFO + and PFO–, *p* < 0.001 for comparison between PFO + and control) and O2 channels (*p* = 0.005 for comparison between PFO + and PFO –, *p* < 0.001 for comparison between PFO + and control), and at 14 Hz on O2 channel (*p* = 0.006 for comparison between PFO + and PFO –, *p* < 0.001 for comparison between PFO + and control) compared to MA patients with PFO – and control group. Whereas the MO patients with PFO + showed an increased alpha band PSD during photic stimulation at 12 Hz on both O1 (*p* = 0.004 for comparison between PFO + and PFO –, *p* = 0.002 for comparison between PFO + and control) and O2 (*p* = 0.001 for comparison between PFO + and PFO –, *p* = 0.001 for comparison between PFO + and control) channels compared to PFO-MO patients and controls. Besides, an increased beta band PSD during photic stimulation at 10 Hz (*p* = 0.007 for comparison between PFO + and PFO –, *p* = 0.008 for comparison between PFO + and control) and 60 Hz (*p* = 0.007 for comparison between PFO + and PFO –, *p* = 0.006 for comparison between PFO + and control) on O1 channel, at 20 Hz (O1 channel *p* = 0.003 for comparison between PFO + and PFO –, *p* = 0.002 for comparison between PFO + and control; O2 channel *p* = 0.006 for comparison between PFO + and PFO –, *p* = 0.005 for comparison between PFO + and control) and 25 Hz (O1 channel *p* = 0.005 for comparison between PFO + and PFO –, *p* = 0.005 for comparison between PFO + and control; O2 channel *p* = 0.006 for comparison between PFO+ and PFO–, *p* = 0.008 for comparison between PFO+ and control) on both O1 and O2 channels are presented in PFO+ MO patients (Figure 2).

### 3.3. Functional connectivity differences between groups

**Figure 3:**
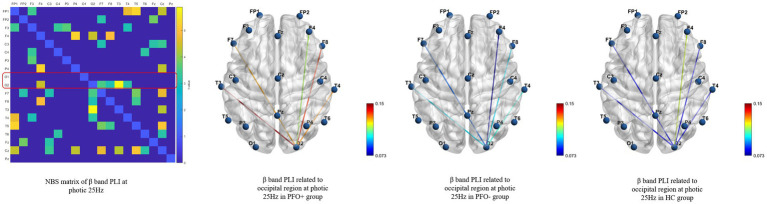
Network-based statistics (NBS) results of the beta band phase lag index (PLI) during photic stimulation among PFO + group (*n* = 35), PFO-group (*n* = 35) and Control group (*n* = 20) during photic stimulation at 25 Hz. The matrix graphs show a significant difference of the beta band PLI between the three groups, whereas the brain connectivity graphs show that the beta band PLI between the occipital region and other regions was increased in the PFO + group compared to the PFO-group.

Functional connectivity revealed several significant differences between PFO+ group, PFO– group and control group by means of NBS method. The beta band PLI presented a significant difference during photic stimulation at 14 Hz (adjusted *p* = 0.021) and 25 Hz (adjusted *p* = 0.042) among three groups. Post-hoc analysis only showed that PFO + group had an increased PLI compared to PFO –group (adjusted *p* = 0.008, Figure 3) during photic stimulation at 14 Hz.

No significant results for subgroup analysis.

### 3.4. Spectrum power associations with migraine characteristics

Previous spectrum power analysis revealed that during photic stimulation, the occipital PSD of the beta band was significantly increased in the PFO + group. On that basis, we correlated the occipital beta band PSD during photic stimulation to migraine clinical characteristics using FDR correction for two channels and seven clinical characteristics.

**Figure 4:**
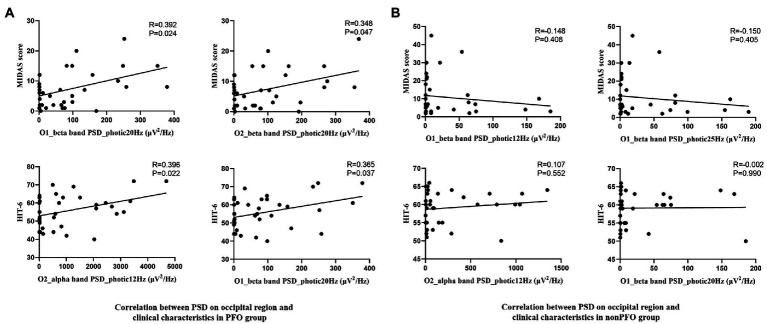
Scatterplot of the beta band PSD of the occipital region during photic stimulation at 20 Hz and the MIDAS score, and alpha band PSD during photic stimulation at 12 Hz, beta band PSD during photic stimulation at 20 Hz and HIT6 score in PFO + (*n* = 35) and PFO– (*n* = 35) group. Positive correlation was found in PFO + group **(A)**, but not in PFO – group **(B)**.

Despite failed to pass the FDR correction, partial correlation analysis showed that the beta band PSD on the O1 channel during photic stimulation at 20 Hz (*p* = 0.037, *R* = 0.365) and the O2 channel at 12 Hz (*p* = 0.022, *R* = 0.396) photic stimulation was prone to positively related to HIT-6 score in the PFO + group. Meanwhile the beta band PSD on both O1 (*p* = 0.024, *R* = 0.293) and O2 (*p* = 0.047, *R* = 0.348) channels during photic stimulation at 20 Hz was prone to positively related to MIDAS score (Figure 4A). In addition, the above PSD indicators were not found to correlate with any clinical characteristics in the patients of PFO– group (Figure 4B).

Functional connectivity analysis indicates that the beta band PLI was significantly increased in the PFO+ group during photic stimulation at 14 Hz. We further investigate the correlations between those different PLIs and migraine clinical characteristics using partial correlation analysis with FDR correction for seven clinical characteristics, but no significant correlation was found.

## 4. Discussion

Using EEG power spectrum analysis and functional connectivity analysis, this study seeks to determine whether PFO influences brain function in migraine patients. The current study found that the alpha and beta band power on the occipital region during photic stimulation at 12-60 Hz was significantly higher in the migraine with PFO group. In terms of functional connectivity analysis, the beta band PLI increased significantly during photic stimulation in the PFO+ group. Furthermore, the beta band power on the occipital region during photic stimulation at 12 and 20 Hz was positively associated with MIDAS and HIT-6 score in migraineurs with PFO.

Several decades have passed since studies emphasizing abnormal brain activity in migraineurs using EEG were conducted (Golla and Winter, 1959; Bjørk et al., 2011; de Tommaso et al., 2014; Shiina et al., 2019). This suggests that cortical excitability may be elevated in migraine patients. Furthermore, migraine with aura exhibited a different function of the occipital cortex compared to migraine without aura (de Tommaso et al., 2013, 2017), indicating that the excitability of the occipital cortex in migraine with aura may be increased. Thus, the increased beta band PSD on the occipital region caused by photic stimulation in the PFO+ group found in this study suggested that the occipital cortex in migraine patients with PFO may be more sensitive to photic stimulus than migraine patients without PFO. Because the beta band activity is generally associated with cortical activation, increased beta band activity is likely to result in abnormal status quo persistence and causes reduced habituation evoked by photic stimulation (Engel and Fries, 2010), which has been supported by numerous previous studies (Bednář et al., 2014; de Tommaso et al., 2014; Coppola et al., 2015; Rauschel et al., 2016; Ambrosini et al., 2017).

Similar to this study, Sevgi et al. (2012) discovered that migraine patients with aura who had large PFOs had increased spectrum power of all frequency bands throughout the entire brain after intravenous microbubble injection, whereas no changes were observed in migraine patients without PFO or PFO patients without migraine, indicating that migraine with aura patients with PFO was more susceptible to microembolism. Their study also discovered that two patients experienced headaches with visual auras shortly after microbubble injection (Sevgi et al., 2012), implying that microembolism-induced EEG changes may serve as a bridge between transient bioelectrical abnormalities and clinical attaches. PSD analysis results aided this study, with a particular emphasis on the occipital region. Although the mechanism of flash stimulation is different from that of microembolism, both of them reflect altered neuro-excitability in migraine patients with PFO, and microembolism likely play an important role between PFO and migraine.

Electroencephalography-measured changes in brain connectivity may be caused primarily by dynamic relationships between cortical and subcortical areas (Friston, 2011). Previous research using EEG connectivity analysis found that in migraine patients, the alpha band synchronization was higher than in healthy controls, while information flow across channels was higher in migraine with aura patients (Bjørk et al., 2011), indicating that thalamocortical dysrhythmia was different in migraine with aura patients than in migraine without aura patients. The beta band PLI was significantly increased in the PFO + group may indicate that the PFO may affect the visual processing network of migraineurs, generalizing it to affect the brain’s processing of sensations.

There was a correlation between occipital beta band PSD during photic stimulation with MIDAS and HIT-6 score in PFO + groups, indicating that the excitability of the occipital cortex may partially represent the severity of headache in migraineurs with PFO. This finding lends credence to the theory that the PFO has a long-term effect on migraine brain activity. In other words, the beta band PSD of occipital regions during photic stimulation may have the potential to present the severity of headaches and predict the effectiveness of therapeutic treatment in migraine patients with PFO. Because the biological basis of changes in the brain network is complex, the lack of correlation between the alpha and beta band PLI and clinical features in the PFO + group may indicate that these network patterns are influenced by other factors. Despite this, this research discovered a distinct brain network pattern in migraine patients with PFO.

We do not know the exact mechanism by which PFO interacts with migraine brain activity, but the findings of this study clearly show that migraine with PFO patients has significantly different brain activity than migraine without PFO patients. The repeated microembolism *via* the right-to-left shunt could be one possible mechanism. PFO may cause paradoxical microembolism, which can result in CSD in mice (Nozari et al., 2010) and cause an attack of headaches. Meanwhile, repeated microembolism may cause chronic hypoxia in the local area that is insufficient to cause an infarction. Given that hypoxia can cause mitochondrial dysfunction in the brain and increase susceptibility to CSD (Gerich et al., 2006; Angelova et al., 2015), and the previous clinical study found that mitochondrial function was reduced in migraine patients (Lodi et al., 2001; Reyngoudt et al., 2011), we hypothesize that migraineurs with PFO’s cortical excitability changes are caused by mitochondrial dysfunction caused by repeated microembolism-induced hypoxia. Besides, resent study aiming at PFO with MA patients has find out that the platelet activation indicators of MA patients with PFO was significantly higher compared to controls, and reduced to normal after PFO closure. Also, the elevated platelet activation may be more controlled by P2Y_12_-blockade rather than by aspirin (Trabattoni et al., 2022). This indicating that the platelet activation may play an important role in PFO and migraine, the abnormal EEG patterns in PFO+ migraineurs may be also due to this.

Although the PREMIUM and PRIMA trials failed to conclude that PFO closure is effective for migraineurs with PFO (Mattle et al., 2016; Tobis et al., 2017), we still cannot say it is completely useless. A recent study developed a nomogram that included migraine with aura, antiplatelet history, and right-to-left shunt at rest had good predictive accuracy for migraine cessation in patients with PFO after PFO closure (Zhao et al., 2020), which means that PFO closure is an effective treatment for at least some PFO migraineurs. This study demonstrates that changes in brain activity during photic stimulation, such as occipital beta band PSD, have the potential to be at least a supplementary marker for predicting the effectiveness of PFO closure for migraineurs with PFO.

However, the findings of this study must be considered in light of its limitations. Firstly, the sample size of this study is small and can only be considered as a pilot study in this field, and future studies with larger sample sizes are needed to clarify the specific effects of PFO on migraine cortical function. Secondly, this study used the cTTE rather than the gold standard cTEE (contrast transesophageal echocardiography) method to identify PFO, so the diagnostic accuracy of PFO is questionable, but the presence of a large right-to-left shunt in all patients is clear. Furthermore, differences in EEG indices were not observed at all frequencies of photic stimulation, particularly the functional connectivity results demonstrated differences in only photic stimulation at 25 Hz, most likely due to more factors and complexities affecting the functional connectivity. The most important factor may be the severity of ischemic foci or cerebral white matter demyelination, which unfortunately was not included in the statistics because of the low percentage of patients included in this study who completed cranial imaging and the low concordance of imaging data. This may reduce the accuracy of the results. In addition, if the EEG of each migraine attack period could be monitored and compared, the specific effect of PFO on brain function in migraine could be better demonstrated. However, this study only included patients with episodic migraine, and it was difficult to collect EEG data during the ictal or peri-ictal period, which is another limitation of this study. Finally, the specific molecular mechanism of the altered EEG pattern in migraine with PFO is only speculated in this study, and further studies could collect biological samples from patients or using animal studies to explore the possible molecular mechanism.

Further research would look into whether these changes in brain activity could be a potential predictor of the effectiveness of PFO closure or P2Y12-blockade, such as Clopidogrel. In addition, the combination of neuroimaging and EEG techniques can complement the methodological limitations of this study and make the results more reliable. Furthermore, animal studies would be another attempt to discover the underlying mechanisms of PFO and migraine, as well as to investigate new therapeutic strategies for migraineurs with PFO.

## Data availability statement

The data analyzed in this study is subject to the following licenses/restrictions: All datasets used and/or analyzed during the current study are available from the first author on reasonable request. Requests to access these datasets should be directed to XL, aluprayz@gmail.com.

## Ethics statement

The studies involving human participants were reviewed and approved by Ethics Committee of Xi’an Jiaotong University First Affiliated Hospital (XJTU1AF2020LSK-226). The patients/participants provided their written informed consent to participate in this study.

## Author contributions

XL study design, manuscript preparation and editing. MW study guidance and manuscript editing. YQ, LW, and CL EEG data acquisition and analysis. YG, YX, and XC clinical data acquisition and analysis. RL ultrasound data acquisition and analysis. GL study guidance and conception, manuscript editing. All authors contributed to the article and approved the submitted version.

## Funding

The present study was supported by Research and Development Funds of Xi’an Jiaotong University First Affiliated Hospital (No. 2021ZYTS-12).

## Conflict of interest

The authors declare that the research was conducted in the absence of any commercial or financial relationships that could be construed as a potential conflict of interest.

## Publisher’s note

All claims expressed in this article are solely those of the authors and do not necessarily represent those of their affiliated organizations, or those of the publisher, the editors and the reviewers. Any product that may be evaluated in this article, or claim that may be made by its manufacturer, is not guaranteed or endorsed by the publisher.

## Footnotes

^1^ http://neuroimage.usc.edu/brainstorm

^2^ http://psychophysiology.cpmc.columbia.edu/Software/CSDtoolbox

^3^ http://www.nitrc.org/projects/bnv/
