# Supplementary material for: The patent foramen ovale may alter migraine brain activity: A pilot study of electroencephalography spectrum and functional connectivity analysis
Source: Front Mol Neurosci. 2023 Mar 7;16:1133303. doi: 10.3389/fnmol.2023.1133303 (PMC10029922; doi:10.3389/fnmol.2023.1133303)
Supplement: Supplementary file 1 [file Table_1.DOCX]

**Supplementary Table 1** Clinical characteristics of Control and MA patients of two migraine groups

|  | Migraine with PFO group | Migraine without PFO group | Control group | Sig. |
| --- | --- | --- | --- | --- |
| N | 11 | 7 | 20 | —— |
| Age (years) | 30.91±9.35 | 31.29±11.53 | 38.40±13.21 | 0.181 |
| male/female | 4/7 | 2/5 | 7/13 | 0.937 |
| Educational qualifications (years) | 12.27±5.26 | 12.43±3.91 | 14.45±2.91 | 0.258 |
| Course (years) | 9.45±7.24 | 16.71±11.38 | —— | 0.115 |
| Attach frequency (attaches/month) | 2.36±3.26 | 1.96±1.34 | —— | 0.763 |
| Attach days (days/month) | 2.37±3.29 | 1.96±1.34 | —— | 0.758 |
| VAS* | 5.73±1.56 | 6.57±1.90 | —— | 0.318 |
| Duration (hours) | 6.18±4.72 | 9.86±9.46 | —— | 0.286 |
| HIT-6* | 55.18±8.53 | 59.86±3.34 | —— | 0.125 |
| MIDAS* (total days) | 3.27±4.22 | 4.57±2.99 | —— | 0.491 |

*: VAS: Visual Analogue Scale; HIT-6: Headache impact test-6; MIDAS: Migraine Disability Assessment Scale.

Continuous variables are expressed as mean ± SD.
