# Supplementary material for: The patent foramen ovale may alter migraine brain activity: A pilot study of electroencephalography spectrum and functional connectivity analysis
Source: Front Mol Neurosci. 2023 Mar 7;16:1133303. doi: 10.3389/fnmol.2023.1133303 (PMC10029922; doi:10.3389/fnmol.2023.1133303)
Supplement: Supplementary file 2 [file Table_2.DOCX]

**Supplementary Table 2** Clinical characteristics of Control and MO patients of two migraine groups

|  | Migraine with PFO group | Migraine without PFO group | Control group | Sig. |
| --- | --- | --- | --- | --- |
| N | 24 | 28 | 20 | —— |
| Age (years) | 36.92±11.29 | 34.26±10.73 | 38.40±13.21 | 0.748 |
| male/female | 0/24 | 5/23 | 7/13 | **0.008** |
| Educational qualifications (years) | 14.54±3.85 | 14.07±3.54 | 14.45±2.91 | 0.876 |
| Course (years) | 9.79±8.75 | 12.27±9.40 | —— | 0.333 |
| Attach frequency (attaches/month) | 3.41±2.68 | 4.89±3.96 | —— | 0.116 |
| Attach days (days/month) | 4.33±2.71 | 5.93±5.00 | —— | 0.167 |
| VAS* | 6.50±1.50 | 6.96±1.64 | —— | 0.293 |
| Duration (hours) | 23.42±22.34 | 19.84±17.18 | —— | 0.517 |
| HIT-6* | 56.13±9.17 | 58.93±4.90 | —— | 0.188 |
| MIDAS* (total days) | 9.08±5.76 | 12.43±11.59 | —— | 0.186 |

* VAS: Visual Analogue Scale; HIT-6: Headache impact test-6; MIDAS: Migraine Disability Assessment Scale.

Continuous variables are expressed as mean ± SD.

Statistically significant P values are shown in bold.
